# The Michelangelo step: removing scalloping and tapering effects in high aspect ratio through silicon vias

**DOI:** 10.1038/s41598-021-83546-w

**Published:** 2021-02-17

**Authors:** Simone Frasca, Rebecca C. Leghziel, Ivo N. Arabadzhiev, Benoît Pasquier, Grégoire F. M. Tomassi, Sandro Carrara, Edoardo Charbon

**Affiliations:** 1 Advanced Quantum Architecture Laboratory (AQUA), École Polytechnique Fédérale de Lausanne (EPFL), 2002 Neuchâtel, Switzerland; 2 Integrated Circuits Laboratory (ICLAB), École Polytechnique Fédérale de Lausanne (EPFL), 2002 Neuchâtel, Switzerland

**Keywords:** Engineering, Electrical and electronic engineering, Electronic devices

## Abstract

We present here, for the first time, a fabrication technique that allows manufacturing scallop free,

non-tapered, high aspect ratio in through-silicon vias (TSVs) on silicon wafers. TSVs are among major technology players in modern high-volume manufacturing as they enable 3D chip integration. However, the usual standardized TSV fabrication process has to deal with scalloping, an imperfection in the sidewalls caused by the deep reactive ion etching. The presence of scalloping causes stress and field concentration in the dielectric barrier, thereby dramatically impacting the following TSV filling step, which is performed by means of electrochemical plating. So, we propose here a new scallop free and non-tapered approach to overcome this challenge by adding a new step to the standard TSV procedure exploiting the crystalline orientation of silicon wafers. Thank to this new step, that we called “Michelangelo”, we obtained an extremely well polishing of the TSV holes, by reaching atomic-level smoothness and a record aspect ratio of 28:1. The Michelangelo step will thus drastically reduce the footprint of 3D structures and will allow unprecedented efficiency in 3D chip integration.

## Introduction

In the last 70 years, the continuous downscaling of semiconductor devices has offered increased device speed and density increases following Moore’s prediction. However, as feature sizes got smaller, down to the current 7 nm (i.e. Intel, Samsung, TSMC), there is a physical limitation in scaling before entering the realm of quantum effects. The semiconductor community has shifted towards 3D integration to achieve higher electrical component density and increased performance. In this context, one of the most promising technologies is the through-silicon via (TSV)^1^.

TSVs are electrical connections etched through silicon that allow for 3D integration and are formed by consecutive steps of etching, insulation, deposition of seed layer, and metallization. The use of TSVs leads to several advantages such as: reduced interconnect length, lower power consumption, increased interconnect density, and ultimately higher functionality and performance, e.g. enhancing signal transmission speed. Faster interconnections between multiple dies and shorter lengths compared to 2D integration also ensure lower capacitive, resistive, and inductive parasitics^2,3^.

TSV fabrication can be categorized based on the steps relative to the complementary metal–oxide–semiconductor (CMOS) fabrication process, such as: via first, if the TSV is formed before the CMOS process, via middle, if the TSV is formed after the devices but before the metal layers, and via last, where the TSV is fabricated after completing all the steps of classical CMOS processes^1^.

The most promising applications for 3D integration using TSV are CMOS image sensors, dynamic random-access memory (DRAM), and heterogeneous integration of different technologies. In 2007, Toshiba released a CMOS Image sensor which was the first commercial product with TSV incorporated in a batch product, and this boosted the growth of 3D integration^4^.

However, the main limitations with the standard TSV fabrication are induced by the so-called scalloping effect, the surface roughness due to the Bosch deep reactive ion etching (DRIE) process^1,5,6^. Scalloping may affect the quality of the seed layer and, in turn, of electroplating. In addition, even when ultra-conformal coating techniques, such as atomic layer deposition, are used for the seed layer, scalloping roughness creates issues related to concentration of stress and electric field in the insulator and barrier layer of the TSV^7^, leading to dielectric breakdown and Cu diffusion during the electroplating step^8^.

In order to eliminate scalloping, we present here a fabrication approach used in the past mostly for optical MEMS application^9–11^, surface smoothening and verticalization by means of KOH etching using <110> oriented silicon wafers: we call this TSV fabrication step the “Michelangelo” step. This smoothening exploits the anisotropic etching properties of potassium hydroxide (KOH) on silicon in order to completely remove the scalloping after Bosch process, polish the internal surfaces of the holes, and then allow a better quality of high aspect ratio holes.

## Material and methods

One of the main new ideas we introduced in our new approach is the exploitation of the silicon structural planes with orientation <111>. We fabricated our holes on 100 mm, 525 μm thick wafers with Si <110> crystalline orientation. These wafers have a crystalline orientation such that one of the <111> plane is perpendicular to the surface, tilted by 35.26° with respect to the main flat. We then designed a mask which has rhomboidal structures, whose edges line up with the <111> plane of the crystalline silicon wafer underneath them. In order to have a clear understanding of the etching process, different hole configurations were analyzed: the rhomboidal hole structures had major diagonal size ranging from 1.5 μm (hence with nominal minor diagonal of 1.06 µm) to 20 μm (with nominal minor diagonal of 14.14 µm), and the pitch between neighboring holes was as small as 1.2 times the major diagonal and as large as 5 times.

**Figure 1:**
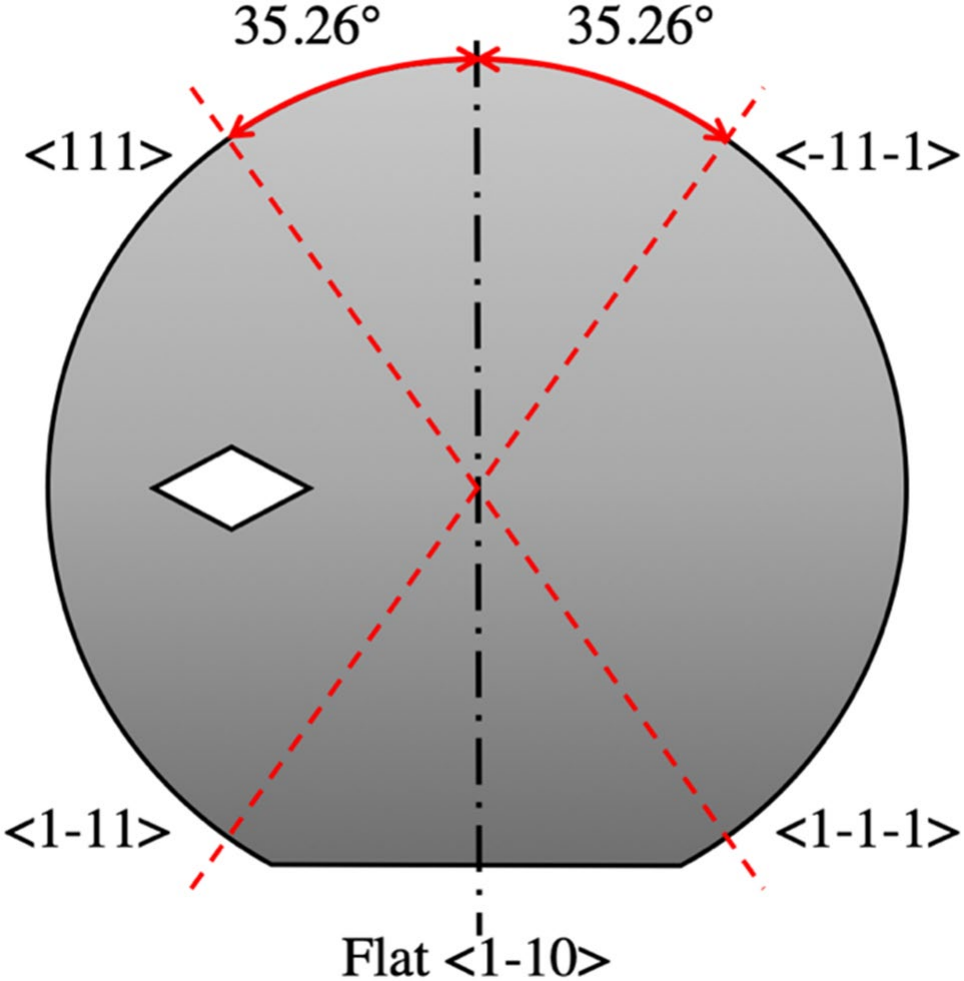
Wafer crystalline orientation of the <111> planes for the <110> -type silicon wafers used for the experiments.

An oxide layer was used as hard mask: wet oxidation was performed to obtain 1.7 μm thick SiO_2_ layer. Despite not the best mask in terms of selectivity, which means a sub-optimal maximum achievable aspect ratio, the SiO_2_ hard mask was thick enough to ensure a good aspect ratio while avoiding the complication and further optimization, which is out of the scope of this paper. The wafer was then coated with 600 nm of AZ ECI 3007 positive tone photoresist and patterned by i-line (λ = 365 nm) photolithography using a Süss MA-6 Gen 3 mask aligner and a chromium mask. The mask was designed in repeated dices fashion (as can be shown from Fig. 1), where both diameter and density factor were swept in each dice. The pattern was then transferred to the SiO_2_ hard mask by means of fluorine plasma etching. The holes in the silicon substrate were etched by means of the Bosch process^12^ using an Alcatel AMS 200 dielectric and silicon etcher system.

The Bosch process is based on alternating depassivation, etch and repassivation steps, using SF_6_ etching plasma and C_4_F_8_ coating gas, pulsed at 6 and 2 s respectively. In our experiments, we achieved a relatively high aspect ratio (AR = 10:1) and lightly tapered holes with evident scalloping roughness on the walls.

**Figure 2:**
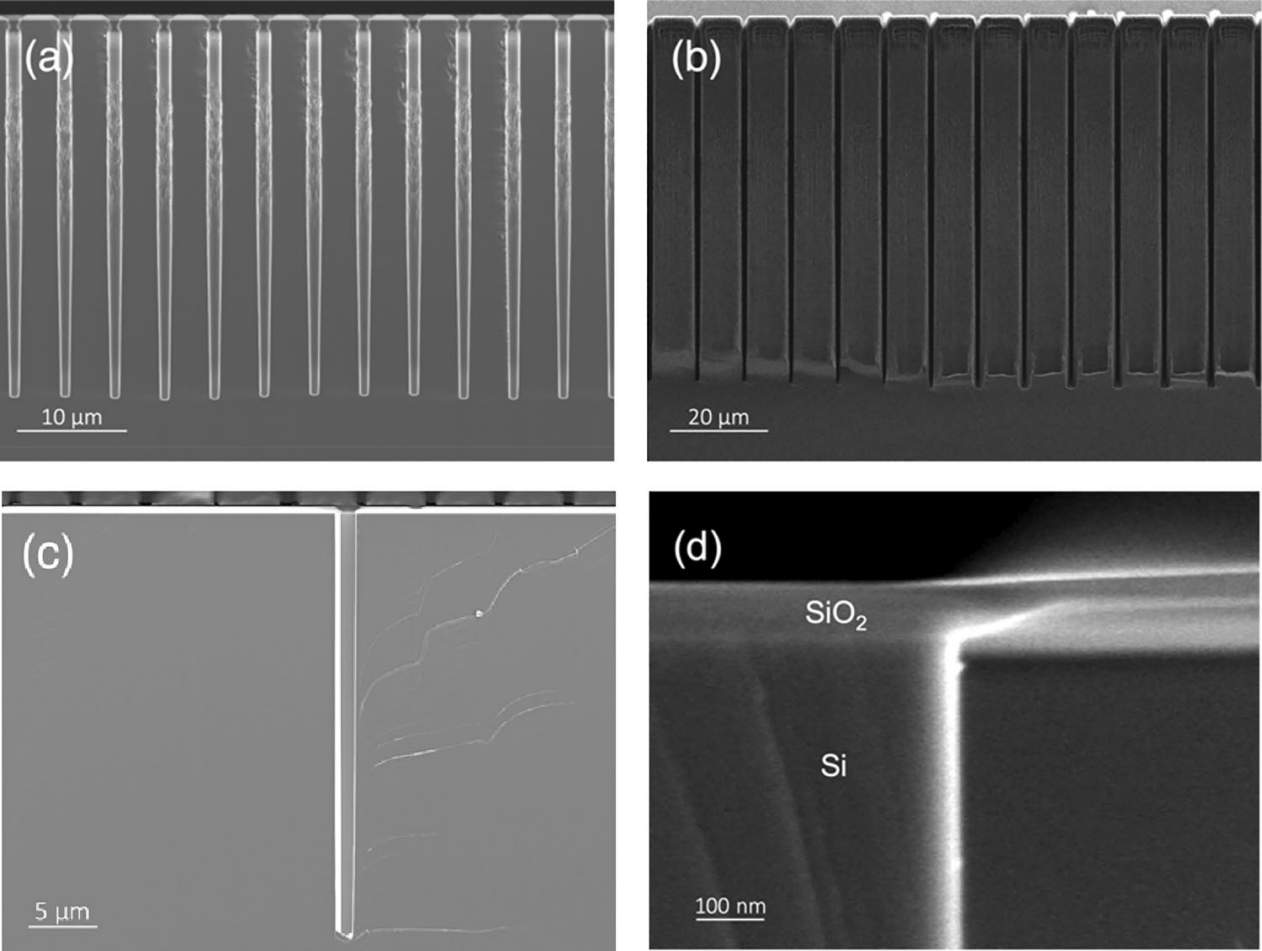
Scanning electron micrograph of TSV holes (**a**) and trenches (**b**) fabricated with the optimized deep reactive ion etching. The width of the hole presents non-negligible bowing effect, going from a minor diagonal size of 1 µm at the top, ~ 1.15 µm in the middle and 0.8 µm at the bottom. Scanning electron micrograph of the Michelangelo-processed TSV hole with an AR of 28:1 (**c**). After KOH etching, bowing in drastically reduced, giving a slightly wider but constant 1.1 µm minor diagonal width at the top and middle of the TSV, dropping to 1 µm at the bottom. Particular of the surface roughness of the TSV after Michelangelo process (**d**).

An alternative and more optimized process has been developed by means of a modified Bosch process. As presented by Xu et al.^13^, we added a cleaning step of O_2_ plasma between passivation and depassivation steps, in order to clean the excess passivation left over after the plasma etching. The addition of this extra step, the use of a lower chamber pressure, higher plasma bias power and lower chamber temperature of 0 °C, as well as the ramping^14^ of process parameters (see Table 1) resulted in a much sharper deep reactive ion etching process, with less rough walls and comparable etching rate to the room temperature process, which reached holes with aspect ratios as high as 28:1 and deep trenches with aspect ratios of 75:1 (see Fig. 2). We did not notice any particular effect in terms of maximum hole depth with respect to different pitch variations. However, we are well aware that there is still room for improvement in our etching fabrication step of the process. Nevertheless, although high aspect ratio is essential for the optimization of a TSV technology process, this particular aspect of the process goes beyond the scope of this work.

**Table 1:** Parameters of the DRIE etching process.

Process	ICP power (W)	Cycle (s)	Gas	Flow rate (sccm)	RF power (W)	Pressure (mbar)
Standard DRIE (20°)						
Passivation	1800	2	C_4_F_8_	300	45	4
Etching	1800	6	SF_6_	300	45	4
Ramping DRIE (0°)						
Passivation	1500	2–3	C_4_F_8_	300	90–110	0.4
Depassivation	1500	1	O_2_	100	90–110	0.4
Etching	1500	3–5	SF_6_	300	90–110	0.4

Parasuraman et al.^14^ show the results of fabrication of deep silicon trenches with an aspect ratio of 160:1 by extreme process optimization and trench sizes as small as 250 nm. The authors show how the achievable aspect ratio increases with smaller feature size. Other interesting results were presented by Owen et al.^15^ achieving an aspect ratio of 97:1 with trenches as large as 3 μm and by Xu et al.^13^ where the authors achieved an aspect ratio of 31.4:1 with 12 μm trenches. Despite close but not higher than these highly optimized values, which require use of ramping parameters of DRIE process, it has to be noted that the optimization of the etching process is important but not the ultimate goal of this work, which aims at presenting a way of removing scalloping and tapering effects in holes after a Bosch process. Also, to be noted is that, differently from previous works, we have optimized the etching parameters for holes and not for trenches, which present a much smaller gas inlet while compared to long trenches. The Knudsen transport model^16^ is even more limiting for holes than it is for trenches, which explains the large differences in achievable aspect ratio using the same process, as confirmed by previous works (see Table 2).

**Table 2:** Comparison of several deep reactive ion etching processes.

Work	Typical size (µm)	Aspect ratio	AR/Size (µm^−1^)	Etching process
H. Li et al. (2018)^17^	100	10:1	0.10	DRIE
Y. Li et al. (2019)^18^	30	10:1	0.33	Modified DRIE
Tillocher et al. (2007)^19^	14	15:1	1.07	Cryo-etching
Shen et al. (2017)^1^	2	15:1	7.5	DRIE
Motoyoshi et al. (2009)^2^	1.4	20:1	14.3	DRIE
Fischer et al. (2012)^20^	20	24:1	1.2	DRIE
This work	1	28:1	28	Modified DRIE

**Figure 3:**
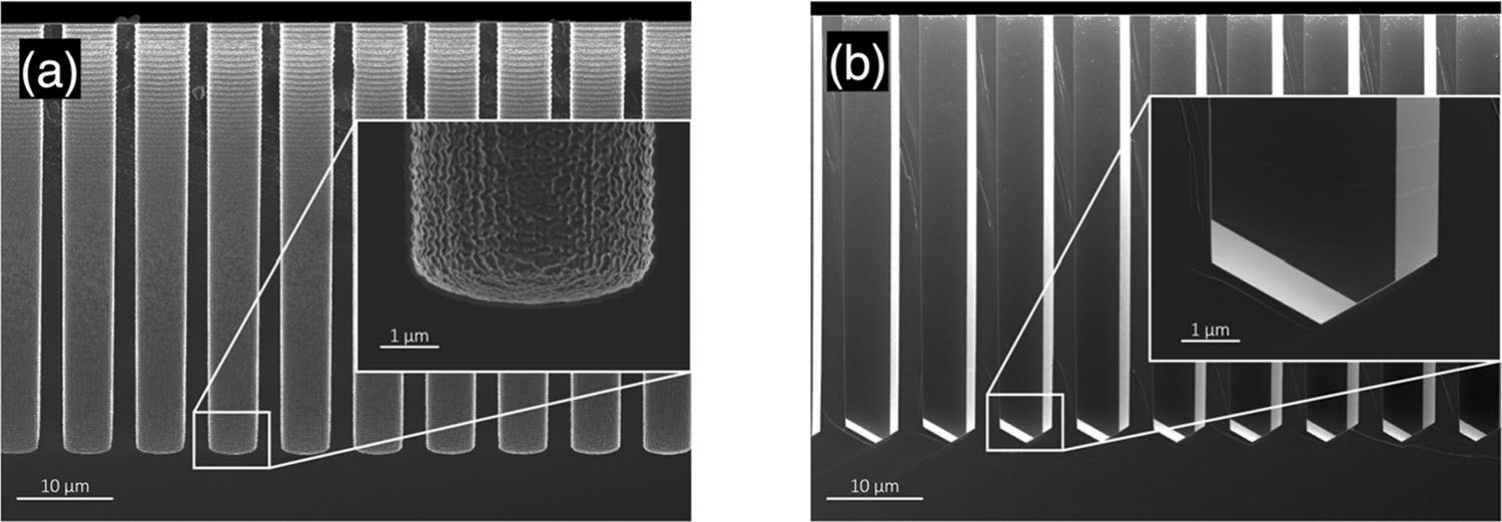
Scanning electron micrograph of TSV holes fabricated without (**a**) and with (**b**) the Michelangelo step. These holes here are 5 µm wide and 40 µm deep: at this aspect ratio, both scalloping and tapering effects are completely removed after immersing the wafer for 60 min in a potassium peroxide bath solution at 40% heated up to 60 °C.

At this step, after the fabrication of the hole with classical methods, we add a surface polishing step consisting of a bath of potassium hydroxide (KOH) solution at 40% heated to 60 °C, which we call the *Michelangelo step*. By aligning the edges of our rhomboidal patterned holes to the <111> plane of the Si <110> wafer, the KOH step only acts on the scalloping roughness and the taper effects of the fabricated holes, while avoiding measurable lateral etching to the walls. On the bottom end of the hole, other <111> planes are met by the KOH, which is what gives the pyramidal shape that can be shown in Fig. 3. Typical etching times range from a few minutes to almost an hour, due to the different aspect ratios and hole sizes. We believe that the difference there might be related to the microfluidic infiltration of the KOH solution, which becomes more relevant for very small holes with large wall roughness. However, as the lateral etching of the vias is negligible after the exposure of the <111> plane, we decided to etch for 45 to 60 min to ensure reproducibility. After cleaning the hole by the "excess" silicon, the wafer is first put into a HCl bath for potassium particle removal, and then into a buffered hydrofluoric acid (BHF) bath at room temperature for the removal of the excess SiO_2_ hard mask.

Metallization follows, with deposition of a barrier layer of 50 nm of alumina (Al_2_O_3_) deposited by atomic layer deposition using a Beneq TFS200. The two reacting precursors, trimethylaluminum (TMAl) and water, are pulsed in the chamber, heated to 200 °C, to allow consecutive single atomic layer depositions of Al_2_O_3_. The seed layer, a thin film of 20 nm of platinum (Pt), was deposited with the same technique in the same machine using as precursors (trimethyl)methylcyclopentadienylplatinum (chemical formula (CH_3_)_3_(CH_3_C_5_H_4_)Pt) preheated at 75 °C and O_2_ reacting in the chamber at 280 °C. Then, to finalize the metallization step, copper electroplating was performed on the samples by an external company (T-Micro, www.t-microtec.com) in non-optimized, standard conditions; the current density used for the Cu electroplating was 0.2 A/dm^2^ for 50 min.

## Results and discussion

We performed TSV fabrication on Si <110> wafers both with and without the proposed wall polishing step. The depth obtained after the deep reactive ion etching was not equal for all hole sizes because the etching rate slows down with increasing aspect ratio: this effect is commonly known as Aspect Ratio Dependent Etching (or ARDE)^21^.

Figure 3 shows the remarkable results obtained with and without our Michelangelo step. It is evident that, together with any effect related to the wall scalloping, also the effect of tapering in the hole disappears after KOH anisotropic etching.

The Michelangelo step allows polishing of vertical walls on every silicon wafer that has vertical <111> direction perpendicular to the plane of the wafer, such as the Si <110> that we used in this work. Proper design of the hole geometry has then to be considered when changing the silicon wafer crystalline orientation.

**Figure 4:**
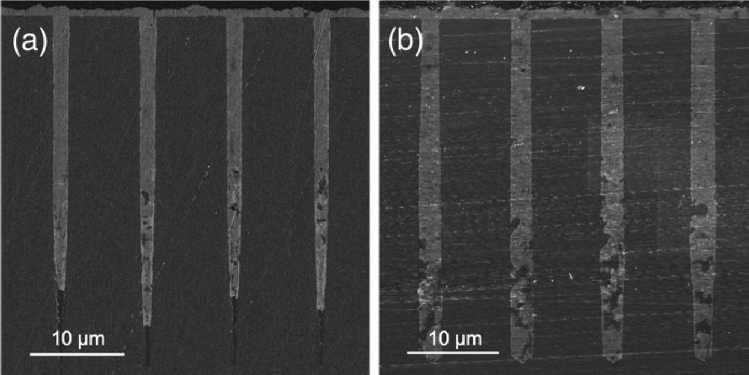
Scanning electron micrograph of finished TSVs fabricated without (**a**) and with (**b**) the Michelangelo step. The two wafers were cut and side-polished to obtain sub-50 nm roughness. Then, for clearer SEM imaging, the sides of the dices were coated with a 13 nm thin carbon film. The apparent difference in aspect ratio between the two images is due to the position of the cut, which was very challenging to align in the very center of the hole in both cases.

The complete removal of scalloping on the side walls of TSVs allows for better fabrication, since the polished walls prevent unwanted effects, such as stress and electric field concentration. Moreover, it helps with the deposition of insulating and seed layers when using deposition techniques different from atomic layer deposition, such as low-pressure chemical vapor deposition (LPCVD). As shown in Fig. 4, the absence of scalloping and tapering effects allows also a better Cu electroplating step, with a more conformal plating in Through-Silicon Vias with high aspect ratio (AR > 15).

In CMOS fabrication one prefers the use of Si <100> wafers for their better silicon dioxide quality. Having a lower silicon atom density at the surface of the silicon–silicon dioxide interface leads to a lower amount of dangling bonds which, in turn, leads to higher carrier mobility. For this reason, the impact of our Michelangelo technology on the CMOS industry may be reduced.

However, this does not apply for other silicon technologies, such as development of top layers of 3D integrated imaging sensors such as APDs, CMOS image sensors (CIS), SPADs and other technologies, or even superconducting circuits technology, such as, for instance, rapid single flux quantum (RSFQ) electronics. Moreover, it is possible to apply a variation of this process while dealing with non-Si technologies, such as InP, InGaAs and other III-V technologies.

## Conclusions

We presented the effect of an additional fabrication step, which allows an extremely effective polishing of the TSV’s walls fabricated on Si <110> wafers. Scalloping removal might have a relevant impact for the yield of high aspect ratio TSVs. Future work will focus on characterization of the electro-thermo-mechanical advantages of such process compared to traditional TSV etching processes.

The name of *Michelangelo step* is related to the famous quote by Michelangelo Buonarroti:

The sculpture is already complete within the marble block, before I start my work.

It is already there, I just have to chisel away the superfluous material.

Indeed, with the final KOH etching step, we just selectively remove all that is superfluous, i.e. the scalloping and the tapering.
